# Challenges of Managing Non-rheumatic Aortic Valve Disorder in a Genetically Susceptible Woman

**DOI:** 10.7759/cureus.37998

**Published:** 2023-04-22

**Authors:** Stephanie Vuong, Alexzandra Hollingworth

**Affiliations:** 1 MSIII, Midwestern University Arizona College of Osteopathic Medicine, Glendale, USA; 2 Surgery, Midwestern University Arizona College of Osteopathic Medicine, Glendale, USA

**Keywords:** cardiovascular genetics, cardio vascular disease, tgf-β1, aortic diseases, aortic valve insufficiency

## Abstract

In this case report, we investigated the potential link between SMAD3/transforming growth factor β (TGF-β) pathway dysregulation and aortic valvular disease. We report a middle-aged female, heterozygous for the R18W novel variant of the SMAD3 gene, with a history of an aortic valve disorder and three aortic valve replacements in a span of 15 years. The patient neither has a history of congenital connective tissue disorders nor any known congenital valvular defects.

The patient had genetic testing for thoracic aortic aneurysm and dissection (TAAD)/Marfan syndrome/related disorders. She was found to be heterozygous for the p.Arg18Trp (R18W) protein variant of the SMAD3 gene (chromosome position 15:67430416), coding DNA c.52 C>T.

Members of the transforming growth factor β (TGF-β) family and their downstream signaling proteins, including SMAD, are important for establishing proper embryogenic development and maintaining adult tissue homeostasis. Investigating the disturbances within the TGF-β signaling pathways may provide insightful knowledge of how genetic factors can cause structural and functional valvular defects.

## Introduction

Aortic valve dysfunctions are categorized as either structural malformations or functional abnormalities. Causes of aortic stenosis may be congenital (intrinsic morphogenesis malformations, extrinsic disruptions, or deformations), degenerative (calcium-fibrin deposits or commissural fusions), or rheumatic [1]. A clinical morphological assessment performed by Waller et al. observed that out of 1973 excised aortic valves, about 91% involved aortic stenosis, and 9% were associated with pure aortic regurgitation [1]. Within the cases of pure aortic regurgitation, 73% were isolated pure aortic regurgitation, and 27% involved the mitral valve [1]. Most cases of aortic stenosis are classified as part of the degenerative aging process; however, Garside et al. found a potential link between improper embryonic valvular development and increased susceptibility to valvular disease in later years [2]. Endothelial-mesenchymal cell transition, proper cell migration, and cell proliferation are key components in proper embryonic cardiac cushion formation and consequential valve development [2].

During early cardiac embryogenesis, endothelial cells within the atrioventricular and conal cushions transform into mesenchymal cells, a process known as endothelial-to-mesenchymal transformation (EndMT) [3]. Mesenchymal cells subsequently migrate into the cardiac jelly, proliferate within the endocardial cushions, and promote the formation of primitive cardiac valves [3]. The successful EndMT process is influenced by many different cytokines, including TGF-β. Dysregulations within the TGF-β pathway may negatively impact proper valve formation. Additionally, multiple studies have shown that increased TGF-β and EndMT are linked to tissue fibrosis in adult years [4].

Cytokines of the TGF-β family are involved in regulating cell renewal and differentiation: playing an important role in embryonic development and adult tissue homeostasis [5]. SMAD proteins are crucial downstream mediators in the TGF-β pathway. They mediate intra-nuclear transcription processes and provide coordination with other cytokine signaling pathways [5]. To date, there are 29 known pathogenic SMAD3 sequence variants, including nonsense, frameshift, and missense mutations. Most of these pathological SMAD3 variants are within exon 6 of the MAD homology domain 2 (MH2) [6]. In this case report, we investigated the potential link between SMAD3/TGF- β dysregulation and aortic valvular disease. We report a middle-aged female, heterozygous for the R18W novel variant of the SMAD3 gene, with a history of aortic valve disorder and three aortic valve replacements in a span of 15 years. She neither has history of congenital connective tissue disorders (i.e., Marfan’s syndrome, Ehlers-Danlos syndrome), nor any known congenital valvular defects (i.e., bicuspid aortic valve). Our patient’s novel SMAD3 mutation has not been listed as a pathological variant; however, her extensive aortic valvular disease history prompts investigation into the TGF-β pathway and its association with cardiac defects.

## Case presentation

A 43-year-old female with a history of two bioprosthetic aortic valve replacements (AVRs) and aortic aneurysm status post aortic grafting, was admitted to the inpatient unit after presenting to the emergency department with severe pleuritic chest pain radiating to her left shoulder, dyspnea, fever, and chills. The patient has an extensive aortic valvular history. When she was 28 years old, she underwent a successful porcine AVR without major complications for severe aortic valve insufficiency (AI) in the presence of a three-leaflet aortic valve with leaflet edge deformity. However, 12 years later, she underwent a second AVR and aortic graft replacement for severe aortic valve stenosis, severe aortic valve insufficiency, degeneration of the prosthetic aortic valve, and a 5.4 cm diameter ascending aortic aneurysm.

The patient had severe chest pain, shortness of breath, and nausea which began in the morning and was constant throughout the day. She self-medicated with high doses of Ibuprofen to minimal relief. Deep inspiration and lying down in a supine position exacerbated the pain. The pain was not preceded by any exertion or trauma. On physical examination, her temperature was 100.2 degrees Fahrenheit, heart rate was 102 beats/min, saturation of peripheral oxygen (SpO2) was 98%, and blood pressure was 126/81 mmHg. She appeared toxic and uncomfortable and had a 3/6 systolic ejection murmur. The rest of her physical examination was normal: her skull was normocephalic and atraumatic, pupils were equal, round, and reactive to light, carotid arteries were clear to auscultation without bruits, lungs were clear to auscultation bilaterally without signs of wheezes or rales, and extremities were non-edematous. An electrocardiogram (EKG) demonstrated normal sinus rhythm at 99 beats/min with left ventricular hypertrophy, but no acute ST elevation to suggest cardiac ischemia. A chest X-ray showed evidence of previous cardiac surgery but no findings of acute congestive heart failure, pneumonia, or pneumothorax. Computed tomography angiography (CTA) of the chest found enhancing soft tissue surrounding the ascending aortic graft suggestive of mediastinitis, aortitis, or infection of the graft, with no aortic dissection. Laboratory findings showed elevated white blood cell (WBC) count at 15.1 K/mcL with neutrophil predominance, C-reactive protein (CRP) at 219.5 mg/L, sedimentation rate at 24 mm/h, and bilirubin at 2.1 mg/dL. Our patient was started on broad-spectrum antibiotic coverage with IV Piperacillin-Tazobactam and Vancomycin after blood cultures were obtained to cover bacterial causes of aortitis and/or endocarditis. A nuclear medicine WBC scan was obtained that was negative. Blood cultures subsequently came back positive for methicillin-sensitive staphylococcus aureus (MSSA) bacteremia, and a brain MRI showed scattered septic emboli with punctate hemorrhages. CTA of the head and neck was obtained that was negative for any mycotic aneurysms.

Computed tomography (CT) of the chest demonstrated the presence of an enlarging ascending aortic peri-graft low-density fluid collection, extensive mediastinal and pericardial enhancement, and organized fluid collection around the ascending thoracic aorta extending from the aortic valve. This was consistent with an aortic graft infection. Because of these findings, she was referred to the surgical team. After routine preparation and continued antibiotic therapy, the patient went to the operating room (OR) for a relatively urgent repeat resection of the ascending graft and redo AVR. During surgery, transesophageal echocardiography (TEE) demonstrated normal aortic valve function, no endocarditis, vegetations, paravalvular leak, or abscess. Left and right ventricular functions were normal, with no significant tricuspid or mitral regurgitation. The patient had a severely inflamed phlegmon surrounding the aortic graft, 6-8 cm in size, and chronic in appearance. Exploration of the mediastinum, aorta, and aortic root was performed and demonstrated a normal aortic root and coronary ostia. The patient successfully underwent a third surgery for a redo sternotomy, redo aortic graft replacement, and redo pericardial tissue AVR. No intraoperative complications were noted. Post-operatively, she struggled with pain, volume overload, anemia, and acute kidney injury (AKI). She was switched to a Daptomycin antibiotic regimen. The patient had two recorded episodes of paroxysmal atrial fibrillation post-operatively, and one episode of paroxysmal atrial flutter with 2:1 block that converted to normal sinus rhythm after IV diltiazem. She was discharged from the hospital with discharge orders for 12 additional weeks of antibiotics, laboratory blood work orders, and health provider follow-up instructions.

After hospital discharge, the patient received genetic testing for thoracic aortic aneurysm and dissection (TAAD)/Marfan syndrome/related disorders. She was found to be heterozygous for the p.Arg18Trp (R18W) protein variant of the SMAD3 gene (chromosome position 15:67430416), coding DNA c.52 C>T. The R18W SMAD3 novel variant is a non-conservative amino acid substitution and has neither been recognized as pathological, nor confirmed to be benign. There were no other pathological variants, deletions, or duplications involving the other genes evaluated (ACTA2, CBS, COL3A1, COL5A2, FBN1, FBN2, FLNA, MED12, MYH11, SKI, SLC2A10, TGFB2, TGFBR1, and TGFBR2).

## Discussion

Members of the TGF-β family and their corresponding downstream signaling proteins, including SMAD, are important for maintaining proper embryological organ development and homeostatic cell proliferation [7]. TGF-β signaling modulates endothelial-mesenchymal cell transition (EndMT), and endothelial cell culture studies demonstrated the role of TGF-β signaling in regulating proper smooth muscle cell differentiation and tubular vessel integrity/arrangement [8]. During early cardiac embryogenesis, polarized endothelial cells within the atrioventricular and conal cushions transform into motile mesenchymal cells [3]. Mesenchymal cells subsequently migrate into the cardiac jelly, proliferate to cellularize the endocardial cushions, and promote the remodeling and formation of our human primitive heart valves [3]. EndMT is characterized by the loss of cobblestone endothelial morphology and cell markers, including vascular endothelial (VE)-cadherin, and the new expression of mesenchymal spindle-shaped morphology and cell markers, including SMA and vimentin [3]. Endothelial-to-mesenchymal transformation is influenced by many different cytokines, most notably, TGF-β. Both in vitro and in vivo studies have shown that TGF-β stimulation is required for endocardial cushion cell transformation in mice [4]. In a study by Yoshimatsu et al., TGF-β2 deficient mice had multiple AV cushion defects. Moreover, inhibition of TGF-β in endocardial cells led to a decrease in EndMT, suggesting that TGF-β inhibition leads to suppression of EndMT [4].

TGF-β, BMP, and NOTCH cytokine signaling pathways have been shown to not only regulate these processes in embryogenesis but also play a role in valve maintenance and remodeling during adult years [2]. Multiple studies have shown that TGF-β associated EndMT is linked to tissue fibrosis. It was demonstrated that cardiac fibrosis is associated with an increase in fibroblast differentiation from endothelial cells, suggesting EndMT plays a role in cardiac fibrosis progression during states of cardiac disease [4]. Patients with sarcomere protein mutation (a-MHC R719W) present with hypertrophic cardiomyopathy as a result of cardiac fibrosis, myocyte enlargement, and impaired ventricular contractility. The mutation activates signals within cells to produce increased amounts of TGF-β [4]. Further evidence also suggests that EndMT not only induces fibroblast proliferation in the heart but also plays a role in intestinal and kidney fibrosis [4].

The TGF-β dysregulation syndromes may manifest as hereditary connective tissue disorders and may be tied to a wide range of nonhereditary cardiovascular defects, including arterial valvular disease, arterial tortuosity, atherosclerosis, and cardiac muscle fibrosis [7, 9]. For instance, studies have shown increased TGF-β levels in Marfan syndrome, hereditary hemorrhagic telangiectasia, and Loeys-Dietz syndromes, in which patients may present with valvular regurgitation, aortic dilation, and vessel aneurysms [7].

In the TGF-β signaling pathway, numerous downstream proteins including a wide variety of SMAD proteins help amplify signaling effects by participating in intranuclear binding and coordination with other cytokine signaling pathways [5]. Exploring the link between TGF-β mediated SMAD or MAPK signaling pathways and valvular disease may provide the insight needed for the treatment and care of valvular defects. Reports investigating the SMAD3 pathway, such as those published by Courtois et al. and Van de Laar et al., have attributed heterozygous SMAD mutations with aneurysms-osteoarthritis syndrome (AOS) or Loeys-Dietz syndrome 3, in which patients can present with arterial aneurysms and tortuosities [6, 8]. Additionally, in a valvular interstitial cell culture study by Das et al., TGF-β1 was found to be upregulated in stenotic valves [10]. An increase in TGF- β1 correlated with an increased reactive oxygen species (ROS) concentration, collagen synthesis, and calcium deposition [10]. Similarly, Song et al. found that TGF β1 and BMP-2 signaling pathways utilize SMAD proteins as downstream mediators to increase pro-osteoclastic activity; signaling dysregulations increase the likelihood of calcific aortic valve disease in human aortic valve interstitial cells (AVICs) [11].

Although this patient has not been confirmed to have known hereditary connective tissue disorders, there may be a correlation with her SMAD 3 variant gene and extensive history of valvular/aortic root disease. To date, there are 29 known pathogenic SMAD3 sequence variants [6]. There is no current literature describing a correlation between our patient's R18W variant with adverse pathology. Of the 2,300 individuals observed in the NHLBI Exome Sequencing Project, the R18W variant was not observed [12]. Likewise, no missense variations of this gene are recorded in the Human Gene Mutation Database [12]. However, the R18W variant is a non-conservative amino acid substitution, which may consequentially impact secondary protein folding, structure, and function.

## Conclusions

Investigating the disturbances within the TGF-β signaling pathways may provide insightful knowledge of how genetic factors can cause structural and functional valvular and arterial wall defects. Highly morbid cardiovascular defects and weakened structural tissue walls, as seen in osteogenesis imperfecta, for instance, are complex to medically manage and damaging to patient welfare. Further study of clinical patterns, genetic correlates, biochemical processes, and environmental factors between TGF-β and cardiovascular disease are warranted to provide insight into future treatment options for managing patients with valvular disease.
